# PD-L1 Expression as a Predictor of Time to Progression in Metastatic Non-Small Cell Lung Cancer Treated with Immune Checkpoint Inhibitors: A Retrospective Single-Center Study

**DOI:** 10.3390/jcm15114191

**Published:** 2026-05-28

**Authors:** Marija Simonovska, Jasmina Djundeva, Bojana Petreska Kapsarov, Julijana Stefanoska, Marina Vukasinovikj Stoleska, Aleksandar Eftimov

**Affiliations:** 1 University Clinic of Radiotherapy and Oncology, 1000 Skopje, North Macedonia; jdjundeva@gmail.com (J.D.); bojanapetreska@gmail.com (B.P.K.); julijanastefanoskamd@gmail.com (J.S.); m.vukasinovik@hotmail.com (M.V.S.); 2 Faculty of Medicine, University of Niš, 18000 Niš, Serbia; 3 Faculty of Medicine, Institute of Pathology, Ss. Cyril and Methodius University, 1000 Skopje, North Macedonia; aleks.eftimov@yahoo.com

**Keywords:** non-small cell lung cancer, PD-L1, immune checkpoint inhibitors, time to progression, objective response rate, real-world study, predictive biomarkers

## Abstract

**Background/Objectives:** Programmed death-ligand 1 (PD-L1) expression is commonly used as a predictive biomarker in metastatic non-small cell lung cancer (NSCLC); however, its clinical utility in real-world settings remains uncertain. This study evaluated the association between PD-L1 expression and treatment outcomes in patients receiving immune checkpoint inhibitors (ICIs). **Methods**: In this retrospective, single-center study, 86 patients with metastatic NSCLC treated with ICIs (pembrolizumab or atezolizumab) between January 2020 and December 2025 were included. PD-L1 expression was assessed using 22C3 or SP263 assays and categorized as <50% and ≥50%. The primary endpoint was time to progression (TTP), while the secondary endpoint was objective response rate (ORR). Survival analyses were performed using Kaplan–Meier estimates, log-rank tests, and Cox proportional hazards models. **Results**: PD-L1 data were available for 78 of the 86 patients. The median age was 66 years (IQR 61–73); 76.7% of patients were male and 65.1% were former smokers. Adenocarcinoma was the predominant histological subtype (47.7%), followed by squamous cell carcinoma (41.9%). The ORR was similar between treatment groups (atezolizumab: 62.5%; pembrolizumab: 64.9%; *p* = 0.84). No statistically significant difference in TTP was observed between PD-L1 <50% and ≥50% groups. Kaplan–Meier analysis showed no statistically significant difference in TTP between PD-L1 groups (log-rank *p* = 0.094), although a numerical trend toward shorter TTP was observed in patients with PD-L1 ≥50%. In multivariate Cox regression analysis, PD-L1 ≥50% was not associated with TTP (hazard ratio [HR] 1.53, 95% confidence interval [CI] 0.77–3.05; *p* = 0.20). No other clinical variables, including smoking status and liver metastases, were significantly associated with outcomes. **Conclusions:** In this limited, retrospective single-center cohort with a heterogeneous treatment mix (chemo-immunotherapy and immunotherapy monotherapy), PD-L1 expression was not significantly associated with treatment response or time to progression. While these results should be interpreted cautiously given the modest sample size and the small number of progression events, they are consistent with broader real-world evidence indicating that PD-L1 expression alone may not reliably stratify clinical benefit in metastatic NSCLC. Larger prospective studies integrating PD-L1 with complementary biomarkers are needed to confirm these observations.

## 1. Introduction

Lung cancer remains the leading cause of cancer-related mortality worldwide, accounting for estimated 1.8–1.9 million deaths annually according to the most recent GLOBOCAN estimates, with non-small cell lung cancer (NSCLC) representing nearly 85% of all cases [1,2]. Despite advances in early detection and treatment, the prognosis of patients with metastatic disease remains poor, with historically limited survival outcomes under conventional chemotherapy-based approaches [1,2]. The introduction of immune checkpoint inhibitors (ICIs), particularly antibodies targeting the programmed cell death protein 1 (PD-1) and its ligand (PD-L1), has significantly transformed the therapeutic landscape of advanced NSCLC. Agents such as pembrolizumab and atezolizumab have demonstrated improved survival outcomes compared with chemotherapy in both first-line and subsequent treatment settings [3,4,5].

These therapies act by restoring anti-tumor immune responses through inhibition of PD-1/PD-L1-mediated T-cell suppression, thereby enabling recognition and elimination of tumor cells [6]. PD-L1 expression, typically assessed using the tumor proportion score (TPS), has been widely adopted as a predictive biomarker for selecting patients for immunotherapy. High PD-L1 expression (≥50%) has been associated with improved response rates and survival outcomes in pivotal clinical trials, particularly in the first-line setting with pembrolizumab monotherapy [3,7]. However, the predictive value of PD-L1 expression remains imperfect and context-dependent, as responses are also observed in patients with low or negative PD-L1 expression, while a substantial proportion of PD-L1-high tumors fail to respond [8,9]. This inconsistency reflects the complex and dynamic nature of tumor–immune interactions and underscores the limitations of PD-L1 as a standalone biomarker.

Moreover, real-world evidence increasingly suggests that the relationship between PD-L1 expression and clinical outcomes may differ from that observed in randomized clinical trials. Heterogeneity in patient populations, treatment regimens, comorbidities, and biomarker assessment methods may influence treatment effectiveness outside controlled trial settings [10,11]. In particular, the correlation between PD-L1 expression and time-to-event outcomes such as progression-free survival (PFS) or time to progression (TTP) remains inconsistent across studies.

Time to progression (TTP), as a progression-focused endpoint, may provide a more reliable measure of treatment efficacy in real-world cohorts where overall survival (OS) data are often immature or confounded by incomplete follow-up and competing risks [12,13]. This is especially relevant in retrospective analyses, where accurate documentation of progression events may be more robust than survival outcomes critical in assessing treatment efficacy in advanced NSCLC [14,15,16].

In North Macedonia, lung cancer screening programs are not currently implemented, and comprehensive national registries with detailed clinical and functional outcome data are lacking. Consequently, most patients are diagnosed at an advanced or metastatic stage, limiting opportunities for early intervention. Immune checkpoint inhibitors have been progressively introduced into routine clinical practice, with atezolizumab becoming available for metastatic NSCLC in 2019, followed by subsequent adoption of other agents. However, real-world treatment outcomes may be influenced by healthcare system factors, including access to diagnostics, treatment availability, and continuity of follow-up. These factors underscore the importance of generating real-world evidence to better understand treatment effectiveness in this setting.

Given these uncertainties, further investigation into the predictive and prognostic role of PD-L1 expression in routine clinical practice is warranted. Lung cancer represents a paradigm of high-level precision oncology, reflecting the complex interplay of molecular alterations, tumor heterogeneity, and dynamic immune interactions. In this study, we performed a retrospective real-world analysis of patients with metastatic NSCLC treated with immune checkpoint inhibitors, aiming to evaluate the association between PD-L1 expression and treatment response, as well as time to progression. By addressing this question, we seek to clarify the practical relevance and limitations of PD-L1 as a predictive biomarker in contemporary clinical practice. Additionally, we explored clinicopathological factors associated with treatment outcomes, including metastatic burden and smoking status.

## 2. Materials and Methods

### 2.1. Study Design and Patient Population

This retrospective, single-center study included 86 patients diagnosed with metastatic non-small cell lung cancer (NSCLC) who were treated with immune checkpoint inhibitors (ICIs) at the University Clinic of Radiotherapy and Oncology in Skopje, North Macedonia, between January 2020 and December 2025.

Inclusion criteria were: histologically confirmed non-small cell lung cancer, available PD-L1 tumor proportion score (TPS), treatment with ICIs (atezolizumab or pembrolizumab), and available follow-up data for response and progression.

Patients harboring activating mutations (e.g., EGFR mutations or ALK rearrangements) and patients with multiple primary malignancies were excluded.

Immune checkpoint inhibitor-related adverse events were not analyzed in this study.

Missing data were not imputed and analyses were performed on available cases.

### 2.2. PD-L1 Assessment

PD-L1 expression was assessed by immunohistochemistry on formalin-fixed, paraffin-embedded tumor tissue samples using validated assays (clone 22C3 pharmDx, Dako/Agilent, Carpinteria, CA, USA and clone SP263, Ventana/Roche, Tucson, AZ, USA) according to manufacturer instructions and institutional protocols at the Institute of Pathology at the Faculty of Medicine, North Macedonia. PD-L1 expression was reported as tumor proportion score (TPS), defined as the percentage of viable tumor cells showing partial or complete membranous staining.

Both assays are widely used and have demonstrated comparable clinical performance in NSCLC.

For statistical analysis, patients were dichotomized into two groups, low expression <50% and high expression ≥50%, based on clinically established thresholds.

This cutoff was selected based on its established clinical relevance as a predictive biomarker for response to immune checkpoint inhibitors and to ensure adequate statistical power for subgroup analysis.

Exploratory analyses using the following PD-L1 categorizations (<1%, 1–49%, ≥50%) were not performed due to limited sample size.

### 2.3. Treatment and Response Evaluation

Patients received standard-of-care treatment, including immunotherapy alone or in combination with chemotherapy, according to institutional protocols.

Tumor response was evaluated using Response Evaluation Criteria in Solid Tumors (RECIST) version 1.1. Best overall response was defined as the best documented response from treatment initiation until disease progression.

Radiological assessments were performed at regular intervals according to institutional practice (typically every 8–12 weeks).

### 2.4. Definition of Time to Progression (TTP)

Time to progression (TTP) was defined as the time from initiation of treatment to the date of radiologically confirmed disease progression.

Patients without documented progression were censored at the date of last follow-up. By definition, TTP considers only radiologically confirmed progression as an event; deaths occurring in the absence of documented radiographic progression were therefore not counted as TTP events and were censored at the date of death. This convention was applied consistently for all patients to preserve the progression-specific nature of TTP as the primary endpoint, as opposed to progression-free survival (PFS), which combines progression and death from any cause. During follow-up, a small number of patients (*n* = 3) died without prior radiographically documented progression; these patients contributed censored observations to the TTP analysis. The implications of this choice (including the potential for informative censoring) are addressed in the limitations part of the Discussion.

### 2.5. Data Collection

Clinical and pathological data were collected from electronic medical records, including: age, smoking status, sex, histological subtype, PD-L1 expression, type of immunotherapy, best response, and dates of treatment initiation and progression.

### 2.6. Statistical Analysis

Statistical analyses were performed using jamovi software (version 2.7; The Jamovi project, Sydney, Australia).

Survival curves were estimated using the Kaplan–Meier method and compared using the log-rank test. Cox proportional hazards regression models were used to evaluate factors associated with TTP. Multivariable models included clinically relevant variables (PD-L1 expression, smoking status, and presence of liver metastases). Given the limited number of progression events (*n* = 35) and the resulting events-per-variable ratio, the multivariable Cox model is acknowledged as exploratory; covariates were pre-specified on clinical grounds (PD-L1 status, smoking status, liver metastases) rather than selected by data-driven procedures, and hazard ratios are reported with 95% confidence intervals to convey precision. Categorical proportions (including ORR and DCR) are reported with 95% Wilson score confidence intervals, and between-group differences in proportions are accompanied by 95% confidence intervals for the absolute risk difference where appropriate.

A *p*-value <0.05 was considered statistically significant.

Model performance was assessed using the concordance index (C-index).

### 2.7. Generative AI Disclosure

Generative artificial intelligence tools were used to assist in language refinement and structuring of the manuscript. All scientific content and interpretations were verified and approved by the authors.

## 3. Results

### 3.1. Basic Patient Characteristics

A total of 86 patients with metastatic NSCLC were included in the analysis. PD-L1 expression data were available for 78 patients, who were included in subsequent subgroup analyses. The median age at diagnosis was 66 years (interquartile range [IQR], 61–73; mean 66.2 ± 8.7 years). The majority of patients were male (76.7%), and most were former smokers (65.1%). Histologically, adenocarcinoma was present in 47.7% of patients, squamous cell carcinoma in 41.9%, NSCLC-NOS in 8.1%, and large cell carcinoma in 2.3%.

At the initiation of immunotherapy, most patients had stage IVA disease (67.4%), while 31.4% had stage IVB disease. Stage data were available for 85 patients. ECOG performance status was 0 in 77.9%, 1 in 16.3%, and 2 in 4.7% of patients; data were available for 85 patients. PD-L1 testing was predominantly performed using the 22C3 assay (82.6%), with a smaller proportion tested using SP263. PD-L1 assay data were available for 76 patients. Patients were dichotomized according to PD-L1 expression into PD-L1 < 50% (*n* = 43) and PD-L1 ≥ 50% (*n* = 35), with missing PD-L1 data in 8 patients. Among patients with available PD-L1 data (*n* = 78), 55.1% had PD-L1 <50% and 44.9% had PD-L1 ≥50%.

Baseline metastatic involvement included the brain (12.8%), liver (12.8%), bone (22.1%), and lung (73.3%). Regarding systemic treatment, 55.8% of patients received atezolizumab and 44.2% received pembrolizumab. All patients were treated in the first-line setting, with 76.7% receiving chemo-immunotherapy and 23.3% receiving immunotherapy monotherapy.

Baseline characteristics were well balanced between the two groups, with no statistically significant differences in age (*p* = 0.5), histological subtype (*p* = 0.3), or type of immunotherapy received (*p* = 0.13). Baseline characteristics are summarized in Table 1.

### 3.2. Treatment Response Analysis

Best overall response data were available for 85 of 86 patients. Among evaluable patients, complete response (CR) was observed in 3 patients (3.5%), partial response (PR) in 51 patients (60.0%), stable disease (SD) in 15 patients (17.6%), and progressive disease (PD) in 16 patients (18.8%). The objective response rate (ORR) was 63.5% (95% CI, 52.8–72.9%), while the disease control rate (DCR) was 81.2% (95% CI, 71.6–88.1%) (Table 2). These findings indicate a high overall response rate in a real-world cohort treated with immune checkpoint inhibitors.

**Figure 1:**
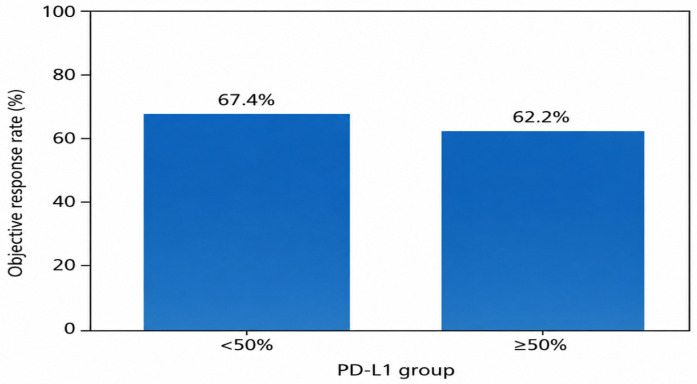
Objective response rate (ORR) according to PD-L1 expression group (<50% vs ≥50%).

Among patients with available PD-L1 and response data (*n* = 78), no significant association was observed between PD-L1 expression and best overall treatment response. In the PD-L1 < 50% group, complete response (CR), partial response (PR), stable disease (SD), and progressive disease (PD) were observed in 4.7%, 65.1%, 14.0% and 16.3% of patients, respectively. In the PD-L1 ≥ 50% group, the corresponding rates were 2.9%, 62.9%, 14.3%, and 20.0%. The distribution of responses did not differ significantly between PD-L1 groups (chi-square *p* = 0.955). Similarly, the objective response rate (ORR) was comparable between the PD-L1 < 50% and PD-L1 ≥ 50% groups (69.8% [95% CI, 54.9–81.4%] vs. 65.7% [95% CI, 49.2–79.2%], Fisher’s exact *p* = 0.809; absolute risk difference, + 4.1%, 95% CI—17.0% to +25.0%), as was the disease control rate (DCR) (83.7% [95% CI, 70.0–91.9%] vs. 80.0% [95% CI, 63.9–90.4%], Fisher’s exact *p* = 0.770) (Table 3). A graphical comparison of ORR between PD-L1 expression groups is presented in Figure 1.

### 3.3. Factors Associated with Objective Response (ORR)

Exploratory analysis of factors associated with ORR did not identify any statistically significant predictors. ORR was comparable between patients treated with atezolizumab (62.5%) and pembrolizumab (64.9%) (*p* = 0.84), between former (62.5%) and current smokers (65.5%) (*p* = 0.78), between adenocarcinoma (65.9%) and squamous cell carcinoma (61.1%) (*p* = 0.67) and between female (65.0%) and male (63.1%) patients (*p* = 1.00). Regarding metastatic burden, patients with liver metastases demonstrated a numerically lower ORR compared to those without liver involvement (45.5% vs. 66.2%, *p* = 0.18), while patients with lung metastases showed a numerically higher ORR (69.8% vs. 45.5%, *p* = 0.07); however, these differences did not reach statistical significance. No meaningful associations were observed for brain or bone metastases. The exploratory analysis revealed that the previously described factors are not significant predictors of treatment response in this cohort, although there was a numerically possible negative signal for liver metastases and a numerically, but non-significant, trend for lung metastases (Table 4).

### 3.4. Survival Analysis

**Figure 2:**
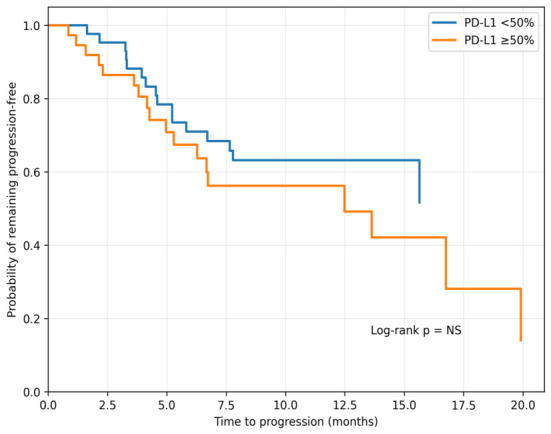
Kaplan–Meier curves for time to progression (TTP) according to PD-L1 expression group (<50% vs ≥50%).

The median follow-up duration was 11.8 months (interquartile range [IQR], 6.2–18.5 months). Time-to-progression (TTP) was analyzed using the Kaplan–Meier method according to PD-L1 expression group. No statistically significant difference in TTP was observed between patients with PD-L1 < 50% and those with PD-L1 ≥ 50% expression (log-rank *p* = 0.094), although a numerical separation of the Kaplan–Meier curves was observed. A total of 35 progression events were observed among 78 patients included in the survival analysis. The restricted mean survival time (RMST) was 33.5 months for the PD-L1 < 50% group and 16.9 months for the PD-L1 ≥ 50% group. Median TTP was not reached in the PD-L1 < 50% group and was 12.5 months in the PD-L1 ≥ 50% group (Figure 2).

### 3.5. Cox Regression Analysis

In univariate Cox regression analysis, PD-L1 expression ≥50% was associated with a non-significant increased risk of progression compared with PD-L1 < 50%; however, this did not reach statistical significance (hazard ratio [HR] 1.65, 95% confidence interval [CI] 0.85–3.23; *p* = 0.14). The concordance index of the univariable Cox model was 0.55, indicating limited discriminative ability of PD-L1 expression alone.

Multivariable Cox regression analysis including PD-L1 expression, smoking status, and presence of liver metastases did not identify any statistically significant predictors of TTP. PD-L1 expression ≥50% remained associated with a numerically increased risk of progression (HR 1.53, 95% CI 0.77–3.05; *p* = 0.20). Similarly, the presence of liver metastases showed a non-significant trend toward worse outcomes (HR 1.54, 95% CI 0.65–3.64; *p* = 0.30), while smoking status was not associated with TTP (HR 0.88, 95% CI 0.42–1.85; *p* = 0.70). These findings suggest that, in this real-world cohort, PD-L1 expression may have limited prognostic value for time to progression.

## 4. Discussion

In this real-world retrospective study of patients with metastatic NSCLC treated with immune checkpoint inhibitors, PD-L1 expression ≥50% was not associated with improved treatment outcomes. Specifically, no statistically significant differences were observed in objective response rate (ORR) or time to progression (TTP) between PD-L1 expression groups, although a consistent numerical trend toward shorter TTP was noted in patients with higher PD-L1 expression. These findings contrast with the results of pivotal randomized clinical trials, such as KEYNOTE-024 and KEYNOTE-042, in which high PD-L1 expression was associated with superior clinical outcomes under pembrolizumab monotherapy [1,2]. However, it is increasingly recognized that the predictive value of PD-L1 is not absolute and may vary depending on clinical context, treatment line, and patient selection [3,4,5].

In the OAK trial, atezolizumab improved overall survival compared with docetaxel in previously treated NSCLC regardless of PD-L1 expression [5], suggesting a broader benefit beyond strictly biomarker-defined populations. Similarly, the IMpower110 study confirmed the efficacy of atezolizumab monotherapy in the first-line setting, particularly in patients with high PD-L1 expression, but also demonstrated overlapping outcomes across PD-L1 subgroups [17]. Furthermore, combination regimens such as those investigated in IMpower150, incorporating chemotherapy and anti-angiogenic therapy with atezolizumab, suggest that the predictive role of PD-L1 may be attenuated in multimodal treatment approaches [18]. These findings support our observation that, in a real-world cohort, PD-L1 expression alone may not fully capture the complexity of treatment response and progression dynamics. One of the key observations in our study is the lack of correlation between PD-L1 expression and ORR. This is consistent with several real-world analyses demonstrating that responses to ICIs occur across all PD-L1 expression levels and that PD-L1 alone is insufficient as a standalone predictive biomarker [6,7,8,9]. Furthermore, a proportion of patients with high PD-L1 expression fail to respond to immunotherapy, highlighting the biological complexity of tumor–immune interactions [10,11].

The absence of a significant association between PD-L1 expression and TTP in our cohort further supports the notion that PD-L1 may have limited prognostic value in heterogeneous real-world populations. In contrast to controlled clinical trials, real-world cohorts include patients with variable performance status, comorbidities, and disease burden, all of which may influence treatment outcomes [19,20,21]. Additionally, differences in PD-L1 assessment methodologies, including antibody clones (e.g., 22C3 vs. SP263) and scoring variability, may contribute to inconsistencies across studies [22,23,24].

From a methodological perspective, the use of two PD-L1 assays (22C3 and SP263) represents a potential source of variability. Although these assays have demonstrated broadly comparable analytical performance in NSCLC, subtle differences in staining characteristics and scoring still exist. Nevertheless, their combined use reflects real-world clinical practice and enhances the generalizability of our findings.

Interestingly, our analysis demonstrated a numerical trend toward shorter TTP in patients with PD-L1 ≥50%, although this did not reach statistical significance. The low concordance index observed in our analysis further supports the limited predictive performance of PD-L1 expression as a single biomarker. While this finding should be interpreted with caution, it may reflect underlying tumor biology, including the presence of aggressive disease phenotypes or immune-evasive mechanisms in certain PD-L1-high tumors [25,26]. Emerging evidence suggests that PD-L1 expression alone does not capture the full spectrum of tumor immunogenicity and that additional factors, such as tumor mutational burden (TMB), immune cell infiltration, and genomic alterations, play a crucial role in determining response to immunotherapy [27,28,29,30].

Our study also explored additional clinical factors associated with treatment outcomes. Although not statistically significant, the presence of liver metastases was associated with a trend toward worse TTP, consistent with previous reports indicating that liver involvement is a negative prognostic factor in patients receiving ICIs [31,32,33,34]. This may be related to the immunosuppressive microenvironment of the liver, which can impair systemic anti-tumor immune responses [35,36]. These observations raise the hypothesis that patients with liver metastases may benefit from more intensive or combinatorial immunotherapeutic approaches, such as dual checkpoint blockade; however, this requires validation in prospective studies.

Similarly, smoking status was not significantly associated with TTP in our cohort. While smoking-related tumors are often characterized by higher mutational burden and increased immunogenicity, the predictive value of smoking status remains inconsistent across studies [37,38,39]. This further underscores the multifactorial nature of immunotherapy response.

Importantly, our findings highlight the limitations of relying solely on PD-L1 expression as a biomarker for treatment selection. Current evidence supports the integration of multiple biomarkers and clinical parameters to better stratify patients and optimize therapeutic strategies [40,41,42,43]. Composite biomarkers incorporating PD-L1, TMB, gene expression profiles, and immune signatures may provide a more accurate prediction of treatment benefit [44,45,46,47].

Another important consideration is the impact of treatment modality. In our cohort, the majority of patients received chemo-immunotherapy, which may diminish the discriminatory role of PD-L1 expression. Several randomized clinical trials such as KEYNOTE-189 and KEYNOTE-407 demonstrated that the addition of chemotherapy to immunotherapy improves outcomes regardless of PD-L1 expression level. This may explain the comparable ORR and DCR observed across PD-L1 subgroups in our study. In this context, PD-L1 may retain value for treatment selection but may be less predictive of treatment response magnitude or duration when combination therapy is used.

From a methodological perspective, the use of TTP as an endpoint represents a strength of our study. In real-world settings, overall survival (OS) data are often immature or confounded by incomplete follow-up, whereas progression events are more reliably captured. TTP therefore provides a clinically meaningful measure of treatment efficacy in retrospective analyses [12,13].

This study has several strengths, including the use of real-world data, the inclusion of both response and time-to-event endpoints, and the focus on a clinically relevant patient population treated according to standard practice. However, several limitations should be acknowledged. First, the retrospective design introduces potential selection bias and limits causal inference. Second, the sample size, particularly within subgroup analyses, may reduce statistical power to detect significant differences. Specifically, with only 35 progression events observed among 78 patients with available PD-L1 data, the events-per-variable ratio in the multivariable Cox model approaches the lower bound of what is generally considered acceptable, increasing the risk of model instability and inflated variance in the estimated hazard ratios. The non-significant trend toward shorter TTP in the PD-L1 ≥50% subgroup should therefore be interpreted cautiously, as the study was not powered to detect modest differences and the confidence intervals around the HR estimates are wide. Type II error and chance fluctuation cannot be excluded, and validation in larger, multi-centre cohorts is warranted before any clinical inference is drawn. Third, variability in imaging intervals and response assessment may influence the accuracy of progression data. Finally, PD-L1 assessment was not standardized across all patients, reflecting real-world clinical practice but introducing potential heterogeneity [14,15,16].

PD-L1 was dichotomized at 50%, which is clinically meaningful but may oversimplify the biological continuum of expression. In addition, TTP was used as the primary endpoint instead of PFS and OS.

Beyond the well-recognised methodological limitations of PD-L1 immunohistochemistry (assay-to-assay variability, observer variability, sampling of a small fraction of the tumor, and the dynamic nature of PD-L1 expression), emerging molecular imaging strategies are beginning to address some of these constraints. Recent preclinical and translational work has shown that radiolabelled small-peptide tracers binding PD-L1, such as the [^68^Ga]Ga-DK223 (PD-L1 PET) tracer evaluated by Mishra et al., can non-invasively quantify total and accessible PD-L1 levels across the whole tumor burden and inform on tumor drug exposure of anti-PD-L1 antibodies [48]. In follow-up work using the [^18^F]DK222 tracer in humanized mouse models of NSCLC and other cancers, PET-derived PD-L1 measurements correlated with treatment-induced changes in PD-L1 expression and with response to anti-PD-1 monotherapy and combination immunotherapy, including in microsatellite-instability-high tumors [49]. While these approaches remain investigational and are not yet clinically validated for treatment selection, they illustrate the direction in which the field is moving: from a static, biopsy-restricted IHC readout toward a dynamic, whole-body quantification of PD-L1 pharmacology. Such developments are conceptually aligned with our observation that PD-L1 expression assessed by conventional IHC, when dichotomized at the 50% cut-off, did not adequately stratify response to ICIs in a real-world setting, and they support the need to integrate spatial, dynamic, and complementary biomarker information into future patient-selection algorithms.

Despite these limitations, our study provides valuable insights into the real-world performance of PD-L1 as a biomarker in metastatic NSCLC. The lack of a strong association between PD-L1 expression and treatment outcomes underscores the need for more comprehensive biomarker strategies and supports a more individualized approach to immunotherapy.

Our findings contribute to the growing evidence that PD-L1 should be interpreted as part of a broader clinical and biological framework rather than as a definitive standalone predictor. Future prospective and real-world studies integrating multiple biomarkers and larger patient cohorts are needed to better define predictors of immunotherapy benefit in NSCLC.

## 5. Conclusions

In this single-centre, retrospective real-world cohort of 86 patients with metastatic NSCLC treated with immune checkpoint inhibitors, PD-L1 expression categorised as the 50% threshold was not significantly associated with objective response or time to progression. Given the modest sample size, the limited number of progression events, the mixed treatment population (chemo-immunotherapy and immunotherapy monotherapy), and the use of two PD-L1 assays, these findings should be interpreted with caution and regarded as hypothesis-generating rather than confirmatory. They are nonetheless consistent with a growing body of real-world evidence suggesting that PD-L1 by immunohistochemistry, when used in isolation and at a single cut-off, does not fully capture the heterogeneity of immunotherapy benefit in advanced NSCLC. Larger, prospective and multi-centre studies that integrate PD-L1 with complementary biomarkers (such as tumor mutational burden, immune-cell infiltrates, gene-expression signatures, and emerging PD-L1 imaging approaches) will be needed to refine patient selection and optimise the use of immune checkpoint inhibitors in this setting.

## Figures and Tables

**Table 1:** Baseline characteristics of the study population (*n* = 86).

Characteristic	Value
**Patient characteristics**	
Number of patients	86
Age at diagnosis, median (IQR), years	66 (61–73)
Age at diagnosis, mean ± SD, years	66.2 ± 8.7
Male, *n* (%)	66 (76.7)
Female, *n* (%)	20 (23.3)
Former smokers, *n* (%)	56 (65.1)
Current smokers, *n* (%)	30 (34.9)
**Tumor characteristics**	
Adenocarcinoma, *n* (%)	41 (47.7)
Squamous cell carcinoma, *n* (%)	36 (41.9)
NSCLC-NOS, *n* (%)	7 (8.1)
Large cell carcinoma, *n* (%)	2 (2.3)
Stage IVA, *n* (%)	58 (67.4)
Stage IVB, *n* (%)	27 (31.4)
ECOG 0, *n* (%)	67 (77.9)
ECOG 1, *n* (%)	14 (16.3)
ECOG 2, *n* (%)	4 (4.7)
**PD-L1 characteristics**	
PD-L1 assay 22C3, *n* (%)	71 (82.6)
PD-L1 assay SP263, *n* (%)	5 (5.8)
PD-L1 < 50%, *n* (%)	43 (55.1)
PD-L1 ≥ 50%, *n* (%)	35 (44.9)
**Metastatic sites**	
Brain metastases, *n* (%)	11 (12.8)
Liver metastases, *n* (%)	11 (12.8)
Bone metastases, *n* (%)	19 (22.1)
Lung metastases, *n* (%)	63 (73.3)
**Treatment characteristics**	
Atezolizumab, *n* (%)	48 (55.8)
Pembrolizumab, *n* (%)	38 (44.2)
First-line treatment, *n* (%)	86 (100)
Chemo-immunotherapy, *n* (%)	66 (76.7)
Immunotherapy monotherapy, *n* (%)	20 (23.3)

Abbreviations: ECOG, Eastern Cooperative Oncology Group; IQR, interquartile range; NSCLC-NOS, non-small cell lung cancer-not otherwise specified; PD-L1, programmed death ligand-1.

**Table 2:** Best overall response in the study population.

Response Category	*n* (%)
Complete response (CR)	3 (3.5)
Partial response (PR)	51 (60.0)
Stable disease (SD)	15 (17.6)
Progressive disease (PD)	16 (18.8)
Objective response rate (ORR)	54 (63.5)
Disease control rate (DCR)	69 (81.2)

**Table 3:** Best overall response according to PD-L1 expression (<50% vs ≥50%).

Response Category	PD-L1 < 50% (*n* = 43)	PD-L1 ≥ 50% (*n* = 35)
Complete response (CR)	2 (4.7)	1 (2.9)
Partial response (PR)	28 (65.1)	22 (62.9)
Stable disease (SD)	6 (14.0)	5 (14.3)
Progressive disease (PD)	7 (16.3)	7 (20.0)
**ORR**	30 (69.8)	23 (65.7)
**DCR**	36 (83.7)	28 (80.0)

**Table 4:** Factors associated with objective response rate (ORR).

Variable	ORR (%)	*p*-Value
**IO agent**		
Atezolizumab (*n* = 48)	30/48 (62.5%)	0.84
Pembrolizumab (*n* = 37 *)	24/37 (64.9%)	
**Smoking status**		
Former (*n* = 56)	35/56 (62.5%)	0.78
Current (*n* = 29 *)	19/29 (65.5%)	
**Sex**		
Female (*n* = 20)	13/20 (65.0%)	1.00
Male (*n* = 65 *)	41/65 (63.1%)	
**Histology**		
Adenocarcinoma (*n* = 41)	27/41 (65.9%)	0.67
Squamous cell carcinoma (*n* = 36)	22/36 (61.1%)	
**Brain metastases**		
Yes (*n* = 11)	6/11 (54.5%)	0.51
No (*n* = 74 *)	48/74 (64.9%)	
**Liver metastases**		
Yes (*n* = 11)	5/11 (45.5%)	0.18
No (*n* = 74 *)	49/74 (66.2%)	
**Bone metastases**		
Yes (*n* = 19)	10/19 (52.6%)	0.32
No (*n* = 66 *)	44/66 (66.7%)	
**Lung metastases**		
Yes (*n* = 63 *)	44/63 (69.8%)	0.07
No (*n* = 22 *)	10/22 (45.5%)	

* Numbers vary due to missing response data; denominators reflect patients with evaluable response.

## Data Availability

The data presented in this study are available upon request from the corresponding author. The data are not publicly available due to patient privacy and ethical restrictions.

## Abbreviations

The following abbreviations are used in this manuscript:

CI	Confidence interval
CR	Complete response
DCR	Disease control rate
ECOG	Eastern Cooperative Oncology Group
HR	Hazard ratio
ICI	Immune checkpoint inhibitor
IHC	Immunohistochemistry
IQR	Interquartile range
NSCLC	Non-small cell lung cancer
ORR	Objective response rate
PD	Progressive disease
PD-1	Programmed cell death protein 1
PD-L1	Programmed death-ligand 1
PFS	Progression-free survival
PR	Partial response
RECIST	Response Evaluation Criteria in Solid Tumors
RMST	Restricted mean survival time
SD	Stable disease
TMB	Tumor mutational burden
TPS	Tumor proportion score
TTP	Time to progression
